# Circulating tumor DNA determining hyperprogressive disease after CAR-T therapy alarms in DLBCL: a case report and literature review

**DOI:** 10.3389/fonc.2023.1283194

**Published:** 2023-11-28

**Authors:** Jiajie He, Rui Zou, Liqing Kang, Lingzi Yu, Peng Wang, Yang Shao, Junheng Liang, Depei Wu, Zhengming Jin, Changju Qu

**Affiliations:** ^1^ Suzhou Medical College of Soochow University, The First Affiliated Hospital of Soochow University, Suzhou, China; ^2^ National Clinical Research Center for Hematologic Diseases, Jiangsu Institute of Hematology, The First Affiliated Hospital of Soochow University, Suzhou, China; ^3^ Institute of Blood and Marrow Transplantation, Collaborative Innovation Center of Hematology, Soochow University, Suzhou, China; ^4^ School of Chemistry and Molecular Engineering, East China Normal University, Shanghai, China; ^5^ Department of Medical Affairs, Shanghai Unicar-Therapy Bio-medicine Technology Co., Ltd, Shanghai, China; ^6^ Department of Medical Affairs, Nanjing Geneseeq Technology Inc., Nanjing, Jiangsu, China

**Keywords:** hyperprogressive disease (HPD), circulating tumor DNA (ctDNA), chimeric antigen receptor T cell therapy (CAR-T), diffuse large B-cell lymphoma (DLBCL), pseudoprogression

## Abstract

Chimeric antigen receptor T-cell therapy (CAR-T) has been widely applied in the clinical practice of relapse/refractory (R/R) diffuse large B-cell lymphoma (DLBCL) due to its promising effects. Hyperprogressive disease (HPD) has gained attention for rapid tumor progression and has become a therapeutic and prognostic challenge. Here, we present a patient who had suffered from several recurrences previously and controlled well with a very small tumor lesion left was infused with CD19/CD22 bispecific CAR-T, with no immune effector cell-associated neurotoxicity syndrome, or cytokine release syndrome observed. However, rapid deterioration, subsequent imaging examination, circulating tumor DNA, and serum biomarkers detection identified HPD. The patient did not respond to salvage treatment and died 40 days after infusion. To our knowledge, only one case of HPD in DLBCL after CAR-T therapy has been reported. This fatal case alarmed the risk of HPD and the ctDNA profile monitoring we used was performed as a non-invasive method to diagnose HPD, providing far-reaching practical instruction for CAR-T therapy.

## Introduction

Diffuse large B-cell lymphoma (DLBCL), a common sub-type of non-Hodgkin’s lymphoma accounting for approximately 30%-40% of cases, is highly invasive, and quite a few patients suffer from relapse/refractory (R/R) disease. Among these cases, the cure rate and overall survival rate are extremely low. The majority are primary lymphomas; others can also be transformed from indolent lymphomas (1). It has been demonstrated that chimeric antigen receptor T-cell (CAR-T) therapy, a topic of long-term discussion, has emerged with promising effects for those who respond poorly to conventional options in multiple types of B cell malignancies (1, 2). Despite great progress in the application of CAR-T therapy in R/R DLBCL, frustration like resistance and relapse, potential toxicities and risks including cytokine release syndrome (CRS), immune effector cell-associated neurotoxicity syndrome (ICANS), long-term hematotoxicity, and other non-negligible adverse events still urgently drive researchers to make improvements and innovations to achieve better safety and efficacy (3).

Hyperprogressive disease (HPD) is now gaining attention for the rapid progression of tumors and is, thus, becoming a therapeutic and prognostic challenge, which can be defined as rapid progression with dramatic acceleration of tumor growth rate in comparison with pretreatment (4, 5) and a time-to-treatment failure of ≤1~2 months instead of benefiting from treatment (6, 7). HPD is observed more likely after immunotherapy associated with immune checkpoint inhibitors like programmed death-1 (PD-1) inhibitor, but its clinicopathological features and exact mechanism remain unclear (8). Relevant studies have reported that the occurrence of HPD may be connected to older age, more prior metastatic sites, higher Eastern Cooperative Oncology Group (ECOG) performance status (PS) score, elevated lactate dehydrogenase (LDH), low programmed death-ligand 1 (PD-L1) expression, and other clinical factors (7, 9).

Here, we report a DLBCL patient who suffered from several recurrences previously and was controlled well, with a very small tumor lesion remaining before CAR-T therapy, but progressed very rapidly and deteriorated quickly after CAR-T therapy. To our knowledge, cases of HPD after DLBCL administrations have rarely been reported. As the second reported case of HPD in DLBCL, circulating tumor DNA (ctDNA) helped determine HPD in this case, demonstrating the potential risks of CAR-T therapy in DLBCL.

## Case report

**Figure 1:**
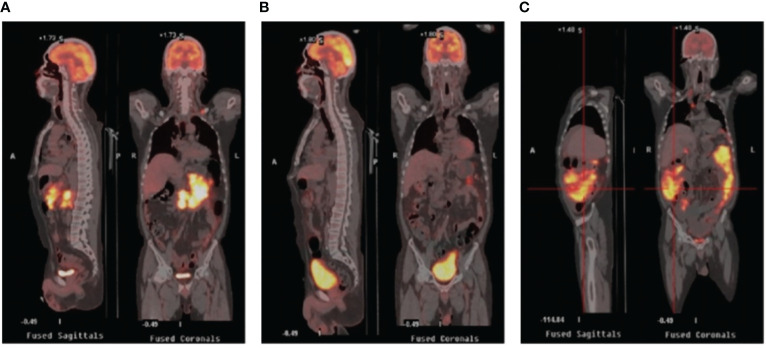
PET/CT images in the periods before CAR-T therapy and after CAR-T therapy. **(A)** Relapse after ASCT showing multiple enlarged lymph nodes with increased glucose metabolism. **(B)** PET/CT after combined targeted therapy showing fewer lesions with smaller volume and reduced metabolism. **(C)** PET/CT after CAR-T infusion showing rapid progression revealing increased lesions and metabolism. PET-CT stands for positron emission tomography-computed tomography; ASCT stands for autologous stem cell transplantation; CAR-T stands for chimeric antigen receptor T-cell.

A 52-year-old man with a history of hepatitis B controlled by entecavir and adefovir dipivoxil was admitted to the local hospital with a progressive enlarging lump in his right groin. Lymph node biopsy confirmed the diagnosis of DLBCL in March 2016. The patient first underwent one cycle of CHOP (cyclophosphamide, doxorubicin, vincristine, and prednisone) chemotherapy followed by five cycles of R-CHOP (rituximab plus CHOP) immunochemotherapy and achieved complete remission (CR). During regular follow-up care, enlargement of the right groin and left cervical lymph nodes was observed in February 2020. Positron emission tomography-computed tomography (PET-CT) showed multiple new hypermetabolic lesions throughout the whole body and tissue biopsy confirmed the relapse of the patient. Subsequently, this patient received nine cycles of CHOPE (etoposide plus CHOP) regimen in 2020, but a new lesion in the left groin appeared 2 months after the treatment, indicating progression of the disease (PD). The treatment was then switched to R-ICE (rituximab plus ifosfamide, carboplatin, and etoposide) immunochemotherapy regimen. After two cycles of R-ICE treatment, further computed tomography (CT) revealed no obvious reduction of the previously enlarged multiple lymph node lesions. He was then treated with OR2 (orelabrutinib, rituximab, and lenalidomide) chemo-free therapy for four cycles starting in April 2021, which led to CR. For long-term benefit, the patient underwent autologous stem cell transplantation (ASCT) in August 2021. Unfortunately, 6 months after ASCT, the patient got PD again with new progression including multiple lymphoid enlargement of the left cervical, left subclavian, mediastinum, and abdominal cavity (**Figure 1A**). Between March and May 2022, two cycles of combined immunochemotherapy therapy (azacitidine, obinutuzumab, and mitoxantrone) were performed and the patient achieved partial remission (PR), with a small and reduced metabolism lesion left according to subsequent PET-CT (**Figure 1B**).

**Figure 2:**
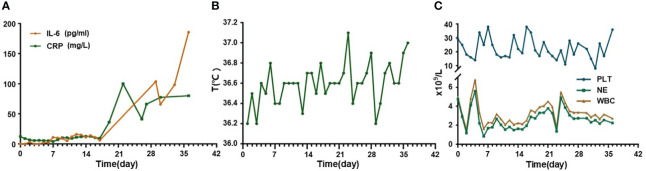
Changes of cytokines, temperature, and hematological examinations in this relapse/refractory diffuse large B cell lymphoma patient. **(A)** Graph showing IL-6 and CRP changes after CAR-T infusion. **(B)** Graph showing temperature changes after CAR-T infusion. **(C)** Graph showing blood cells level changes after CAR-T infusion. CAR-T stands for chimeric antigen receptor T-cell; IL-6 stands for interleukin (IL)-6; CRP stands for C-reactive protein.

Since a series of ineffective therapeutic regimens had been administered with repeated relapse of disease, we proposed CAR-T therapy. FC regimen (fludarabine 30mg/m^2^ qd d1-3, cyclophosphamide 300mg/m^2^ qd d1-3) was used as a lymphodepletion regimen and was started from July 1 to July 3, 2022. Subsequently, CD19/CD22 bispecific CAR-T cells were infused at an escalation dose of 10%, 30%, and 60% from July 6 to July 8, 2022, with a total dose of 1.0x10^7^/kg. Serum interleukin (IL)-6 level showed a slight fluctuating upward trend and rose less than twice compared to the level before infusion. C-reactive protein (CRP) level decreased during the first several days, followed by a slight increase, and the overall level persistently remained below the pre-infusion level (**Figure 2A**). During the whole treatment, no fever, signs of oxygen deficiency, low blood pressure, or neurological symptoms were observed, ruling out CRS and ICANS (**Figure 2B**), but severe myelosuppression had persisted after CAR-T infusion (**Figure 2C**), calling for continued promotion of hematopoietic therapy and component transfusion.

**Figure 3:**
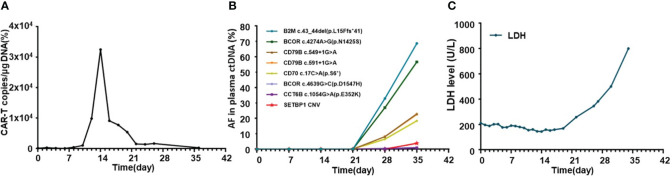
Dynamic profiling of CAR-T copies, circulating tumor DNA, and LDH after CAR-T infusion in this relapse/refractory diffuse large B cell lymphoma patient. **(A)** Volatility curve of CAR-T copies at the indicated time points after CAR-T infusion. **(B)** Line chart showing AF changes and mutations of circulating tumor DNA after infusion. **(C)** Graph showing the changes in LDH level after CAR-T infusion. CAR-T stands for chimeric antigen receptor T-cell; LDH stands for lactate dehydrogenase; AF stands for allele frequency.

The general trend of absolute CAR-T copies in peripheral blood was upward at first and reached a peak of 3.25x10^4^/μg DNA on day 15, while a downward trend was observed in the following days. The overall level of CAR-T copies maintained between 6.12x10^1^/μg DNA and 3.25x10^4^/μg DNA within 1 month after infusion, reflecting relatively low and transient CAR-T proliferation (**Figure 3A**). One month after CAR-T cell infusion, PET-CT (**Figure 1C**) revealed an unexpected enlargement of multiple lymph nodes throughout the whole body, including in the bilateral clavicle area, mediastinum, right internal breast, pelvic cavity, etc., with increased metabolism. In the course of the CAR-T therapy, we simultaneously extracted circulating tumor DNA (ctDNA) from the blood plasma of this patient and measured the levels of ctDNA as previously reported (10) once a week after CAR-T infusion, aiming to further evaluate the efficacy. Generally, targeted next-generation sequencing of ctDNA was performed in a testing laboratory, which was accredited by Clinical Laboratory Improvement Amendments (CLIA) and College of American Pathologists (CAP) (Nanjing Geneseeq Technology Inc., Naning, China). Libraries were constructed using the KAPA Hyper DNA Library Prep Kit (KAPA Biosystem, KK8504). The probes for targeted sequencing covered exons and selected introns of 475 leukemia- and lymphoma-related genes. The library was sequenced as paired 150-bp reads on Illumina HiSeq 4000, and the depth of coverage of the NGS panel was set as 5000x. Single nucleotide variants and short insertions/deletions were identified by VarScan2 2.3.9. Copy number variations were detected using in-house-developed software. Finally, the Delly fusion calling tool was used to identify the number of chimeric reads (sequencing paired ends mapped to different genes) and split reads (spanning a fusion breakpoint) from the targeted DNA-seq data. Eventually, dynamic profiling of ctDNA performed undetectable allele existing on day 0, 7, 14, and 21 after CAR-T infusion but, unexpectedly, allele frequencies were spotted on day 28 and day 35, manifesting a significant upward trend and a continuous progressive elevation of ctDNA allele frequencies. Furthermore, new gene alterations of ctDNA, including CCT6B and SETBP1 were first detected on day 35 (**Figure 3B**). We also monitored the levels of LDH, a marker of tumor burden, observing a relatively synchronous increase as shown in **Figure 3C**.

**Figure 4:**
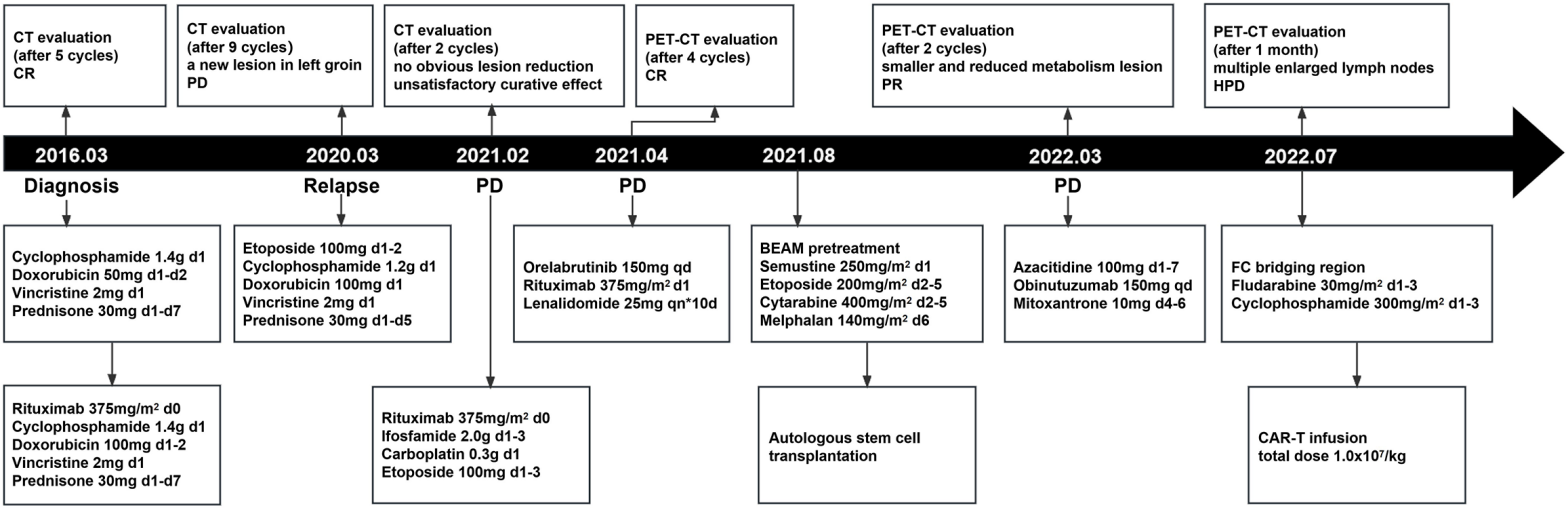
The whole process of multiline therapies and disease status of the patient after he was diagnosed with DLBCL. CT stands for computed tomography; PET-CT stands for positron emission tomography-computed tomography; ASCT stands for autologous stem cell transplantation; CAR-T stands for chimeric antigen receptor T-cell.

Then, the patient was treated with lenalidomide as a salvage treatment. Unfortunately, the patient got no response and developed rapid clinical deterioration during hospitalization and died 40 days after CAR-T therapy (**Figure 4**).

## Discussion

HPD after CAR-T therapy in patients with R/R DLBCL has not yet been widely reported. Our case shows an R/R DLBCL patient who achieved PR before CAR-T therapy but got HPD with widespread lesions 1 month after infusion. This case rang an alarm that not all patients can benefit from CAR-T therapy, and identifying the risk factors driving this regrettable outcome may be broadly informative for DLBCL patients.

HPD has recently raised widespread concerns about the rapid progression of the disease in the post-treatment period. Several existing studies have discussed the potential biological mechanisms of HPD. It seems to be an immunological phenomenon in which multiple factors are involved. The expression of immune resistance mediators induced by the interaction between tumor cells and specific lymphocytes promotes tumor proliferation and invasion. According to the available reports, HPD often occurs after using immune checkpoint inhibitors treatment, especially under anti-PD-1 blocking antibodies that were dramatically effective in malignant tumors, including R/R lymphoma (8). Some *in vitro* studies have shown that overexpressing PD-1 in tumor cells might reduce their viability, while PD-1 blockade was related to increased viability (11). N Nora Bennani et al. reported that 4 of 12 enrolled patients with peripheral T-cell lymphomas developed HPD soon after nivolumab injection, a kind of anti-PD-1 blocking antibodies (6). Daniel A. Rauch et al. reported that all three patients with adult T-cell leukemia/lymphoma enrolled in clinical trial NCT02631746 experienced HPD after infusion of nivolumab. Further analysis revealed the acute dysregulation of gene function (8). Regulatory T lymphocytes also affected the occurrence and development of HPD (11). Related studies have shown the clinical characteristics of HPD and conducted statistical analysis of its risk factors, though there is still a lack of definitive evidence (7, 9, 12).

Pseudoprogression was one of the important manifestations characterized by local inflammatory cell infiltration and immune activation in lesions after immunotherapy, which was hard to distinguish with HPD through imaging examinations. However, pseudoprogression usually suggested better responses and prognosis clinically, while HPD needed further salvage therapy and represented poor prognosis (13). To distinguish between HPD and pseudoprogression, lesion biopsy was valuable. Furthermore, imaging detection, especially 18F-FDG PET-CT, which was important in lymphoma staging, treatment monitoring, and prognostic assessment, also played a significant role in the diagnosis of HPD. Limeng He et al. reported that PET-CT detected HPD 35 days after CAR-T therapy in a 65-year-old woman with R/R DLBCL, and further pathological examination confirmed HPD diagnosis in this case (14), which was also the first and only reported HPD DLBCL case after CAR-T therapy in the literature. Regrettably, lesion biopsy was limited in our patient due to lower platelet counts and his extremely poor general condition. As an invasive method, it might have led to more serious complications at that moment. According to some retrospective evaluations in the relevant literature, ctDNA level and sequencing, which were gradually applied to diagnosis and therapeutic monitoring, may also provide auxiliary clues to predict and distinguish HPD from pseudoprogression (10, 13). Plasma ctDNA detection was an analysis of DNA fragments from the tumor, which helped to observe the heterogeneity of tumors, thus clinically guiding targeted therapy. Compared to the limitations of invasive damage of biopsy, ctDNA was more convenient and efficient for dynamic continuous monitoring in the course of treatment (15). We detected and analyzed ctDNA from the blood plasma of this patient once a week after CAR-T therapy. We found a significant upward trend and a continuous progressive elevation of ctDNA allele frequencies after CAR-T therapy, while at that time, CAR-T copies continued to descend and LDH levels changed to sharply increase, providing clues of the correlation between them (**Figure 3**). New gene alterations of ctDNA including CCT6B and SETBP1, which were first detected on day 35, were reported to be associated with rapid progression and poor prognosis in hematologic tumors according to some literature (16). Increased ctDNA levels and newly emerged mutations confirmed HPD instead of pseudopregression in this patient. In addition, the rapid increase of LDH, a marker of tumor burden and rapid clinical deterioration also supported HPD to some auxiliary extent **Figure 4**.

So far, the efficacy of CAR-T therapy has been confirmed through clinical practice, especially in hematological tumors (1, 2). For patients with R/R DLBCL, the efficacy of multiline therapy was limited, while CAR-T therapy was proven to have a high overall remission rate in clinical practice during recent years. However, only 40% of patients benefit from CAR-T therapy with good survival. Failures after CAR-T therapy usually occur early after infusion. Laetitia Vercellino et al. identified ECOG PS≥2, elevated LDH at the time of decision (TD) with eligibility for CAR-T therapy and extranodal sites ≥2, elevated CRP and total metabolic tumor volume (TMTV) >80 mL at the time of treatment (TT), with lymphodepletion and CAR-T infusion as risk factors to early progression through multivariate analyses of 116 patients who underwent CAR-T therapy (17). In our case, elevated LDH at TD and elevated CRP at TT were observed, which may confer to HPD. Further, Chelsea C Pinnix et al. reported that poor prognosis was associated with patients requiring bridging therapy. In this case, bridging therapy was given concerning high tumor burden. Though bridging therapy decreased tumor burden, which led to lower CRS/ICANS incidences, it may also cause tumor antigen loss, which blocks the proliferation of CAR-T cells *in vivo*. In this case, the patient showed no CRS/ICANS occurrence, but detection of CAR-T copies proliferation was relatively low and transient, which may, eventually, confer to HPD. Thus, future studies are necessary to identify which or whether bridge therapy should be applied to improve CAR-T therapy outcomes in the future (18). Furthermore, the patient was in severe myelosuppression due to multiline therapies and high-intensity treatment including ASCT and CAR-T therapy. While Myelosuppression, one of the major adverse events (19) during CAR-T therapy, might also confer to the occurrence of HPD. Prolonged myelosuppression with blood cytopenia led to an immunity effect of immunosuppression and had the potential to induce HPD.

In summary, this case report took non-invasive ctDNA into consideration to identify HPD and emphasized the potential risks of HPD post CAR-T therapy. However, this was also a single case, which revealed the HPD phenomenon once again, more clinical data are needed to further confirmation. Though CAR-T therapy has great potential for future progress in clinical application, it is necessary to take HPD and its effect on prognosis into consideration when managing DLBCL in future treatment options.

## Data availability statement

The original contributions presented in the study are included in the article/supplementary material. Further inquiries can be directed to the corresponding authors.

## Ethics statement

Written informed consent was obtained from the patient for publication of this case report and any accompanying images.

## Author contributions

JH: Data curation, Formal analysis, Writing – original draft. RZ: Data curation, Formal analysis, Writing – original draft. LK: Data curation, Methodology, Writing – original draft. LY: Supervision, Writing – review & editing. PW: Data curation, Methodology, Writing – original draft. YS: Methodology, Writing – original draft. JL: Methodology, Writing – original draft. DW: Conceptualization, Validation, Writing – review & editing, Supervision. ZJ: Conceptualization, Formal analysis, Supervision, Validation, Writing – review & editing. CQ: Conceptualization, Formal analysis, Project administration, Resources, Supervision, Writing – original draft, Writing – review & editing.

## References

[1] Abramson JS. Anti-CD19 CAR T-cell therapy for B-cell non-hodgkin lymphoma. Transfus Med Rev (2020) 34(1):29–33. doi: 10.1016/j.tmrv.2019.08.003 31677848

[2] Ying Z Yang H Guo Y Li W Zou D Zhou D. Relmacabtagene autoleucel (relma-cel) CD19 CAR-T therapy for adults with heavily pretreated relapsed/refractory large B-cell lymphoma in China. Cancer Med (2020) 10(3):999–1011. doi: 10.1002/cam4.3686 33382529 PMC7897944

[3] Rejeski K Perez A Sesques P Hoster E Berger C Jentzsch L. CAR-HEMATOTOX: a model for CAR T-cell–related hematologic toxicity in relapsed/refractory large B-cell lymphoma. Blood (2021) 138(24):2499–513. doi: 10.1182/blood.2020010543 PMC8893508 34166502

[4] Kanjanapan Y Day D Wang L Al-Sawaihey H Abbas E Namini A. Hyperprogressive disease in early-phase immunotherapy trials: Clinical predictors and association with immune-related toxicities. Cancer (2019) 125(8):1341–9. doi: 10.1002/cncr.31999 30768786

[5] Adashek JJ Subbiah IM Matos I Garralda E Menta AK Ganeshan DM. Hyperprogression and immunotherapy: fact, fiction, or alternative fact? Trends Cancer (2020) 6(3):181–91. doi: 10.1016/j.trecan.2020.01.005 PMC9726601 32101722

[6] Bennani NN Kim HJ Pederson LD Atherton PJ Micallef IN Thanarajasingam G. Nivolumab in patients with relapsed or refractory peripheral T-cell lymphoma: modest activity and cases of hyperprogression. J Immunother Cancer (2022) 10(6):e004984. doi: 10.1136/jitc-2022-004984 35750419 PMC9234908

[7] Liu J Wu Q Wu S Xie X. Investigation on potential biomarkers of hyperprogressive disease (HPD) triggered by immune checkpoint inhibitors (ICIs). Clin Transl Oncol (2021) 23(9):1782–93. doi: 10.1007/s12094-021-02579-9 33847923

[8] Rauch DA Conlon KC Janakiram M Brammer JE Harding JC Ye BH. Rapid progression of adult T-cell leukemia/lymphoma as tumor-infiltrating Tregs after PD-1 blockade. Blood (2019) 134(17):1406–14. doi: 10.1182/blood.2019002038 PMC6839957 31467059

[9] Kim SR Chun SH Kim JR Kim SY Seo JY Jung CK. The implications of clinical risk factors, CAR index, and compositional changes of immune cells on hyperprogressive disease in non-small cell lung cancer patients receiving immunotherapy. BMC Cancer (2021) 21(1):19. doi: 10.1186/s12885-020-07727-y 33402127 PMC7786505

[10] Wang X Su W Gao Y Feng Y Wang X Chen X. A pilot study of the use of dynamic analysis of cell-free DNA from aqueous humor and vitreous fluid for the diagnosis and treatment monitoring of vitreoretinal lymphomas. Haematologica. (2022) 107(9):2154–62. doi: 10.3324/haematol.2021.279908 PMC9425330 35142151

[11] Frelaut M du Rusquec P de Moura A Le Tourneau C Borcoman E. Pseudoprogression and hyperprogression as new forms of response to immunotherapy. BioDrugs (2020) 34(4):463–76. doi: 10.1007/s40259-020-00425-y 32394415

[12] Chen Y Hu J Bu F Zhang H Fei K Zhang P. Clinical characteristics of hyperprogressive disease in NSCLC after treatment with immune checkpoint inhibitor: a systematic review and meta-analysis. BMC Cancer (2020) 20(1):707. doi: 10.1186/s12885-020-07206-4 32727409 PMC7392646

[13] Ruff A Ballard HJ Pantel AR Namoglu EC Hughes ME Nasta SD. 18F-fluorodeoxyglucose positron emission tomography/computed tomography following chimeric antigen receptor T-cell therapy in large B-cell lymphoma. Mol Imaging Biol (2021) 23(6):818–26. doi: 10.1007/s11307-021-01627-8 PMC8578305 34231105

[14] He L Deng Y Deng Y Xie H Zhang W. Early hyperprogression of lymphoma detected by 18F-FDG PET/CT after chimeric antigen receptor T-cell therapy. Clin Nucl Med (2023) 48(3):256–8. doi: 10.1097/RLU.0000000000004543 36634320

[15] Cheng ML Pectasides E Hanna GJ Parsons HA Choudhury AD Oxnard GR. Circulating tumor DNA in advanced solid tumors: Clinical relevance and future directions. CA Cancer J Clin (2021) 71(2):176–90. doi: 10.3322/caac.21650 33165928

[16] Carratt SA Kong GL Curtiss BM Schonrock Z Maloney L Maniaci BN. Mutated SETBP1 activates transcription of Myc programs to accelerate CSF3R-driven myeloproliferative neoplasms. Blood (2022) 140(6):644–58. doi: 10.1182/blood.2021014777 PMC9373012 35482940

[17] Vercellino L Di Blasi R Kanoun S Tessoulin B Rossi C D’Aveni-Piney M. Predictive factors of early progression after CAR T-cell therapy in relapsed/refractory diffuse large B-cell lymphoma. Blood Adv (2020) 4(22):5607–15. doi: 10.1182/bloodadvances.2020003001 PMC7686887 33180899

[18] Pinnix CC Gunther JR Dabaja BS Strati P Fang P Hawkins MC. Bridging therapy prior to axicabtagene ciloleucel for relapsed/refractory large B-cell lymphoma. Blood Adv (2020) 4(13):2871–83. doi: 10.1182/bloodadvances.2020001837 PMC7362355 32589728

[19] Rejeski K Kunz WG Rudelius M Bücklein V Blumenberg V Schmidt C. Severe Candida glabrata pancolitis and fatal Aspergillus fumigatus pulmonary infection in the setting of bone marrow aplasia after CD19-directed CAR T-cell therapy – a case report. BMC Infect Dis (2021) 21:121. doi: 10.1186/s12879-020-05755-4 33509115 PMC7841988

